# 532 The Impact of a Clinical Pharmacist on Medication Management in an Adult Burn Clinic

**DOI:** 10.1093/jbcr/irad045.129

**Published:** 2023-05-15

**Authors:** Allison Boyd, Todd Walroth, Katherine Meadows, Leigh Spera, Brett Hartman

**Affiliations:** Eskenazi Health, Indianapolis, Indiana; Eskenazi Health, Indianapolis, Indiana; Eskenazi Health, Indianapolis, Indiana; Indiana University School of Medicine/Eskenazi Health, Indianapolis, Indiana; Indiana university/Eskenazi Health, Indianapolis, Indiana; Eskenazi Health, Indianapolis, Indiana; Eskenazi Health, Indianapolis, Indiana; Eskenazi Health, Indianapolis, Indiana; Indiana University School of Medicine/Eskenazi Health, Indianapolis, Indiana; Indiana university/Eskenazi Health, Indianapolis, Indiana; Eskenazi Health, Indianapolis, Indiana; Eskenazi Health, Indianapolis, Indiana; Eskenazi Health, Indianapolis, Indiana; Indiana University School of Medicine/Eskenazi Health, Indianapolis, Indiana; Indiana university/Eskenazi Health, Indianapolis, Indiana; Eskenazi Health, Indianapolis, Indiana; Eskenazi Health, Indianapolis, Indiana; Eskenazi Health, Indianapolis, Indiana; Indiana University School of Medicine/Eskenazi Health, Indianapolis, Indiana; Indiana university/Eskenazi Health, Indianapolis, Indiana; Eskenazi Health, Indianapolis, Indiana; Eskenazi Health, Indianapolis, Indiana; Eskenazi Health, Indianapolis, Indiana; Indiana University School of Medicine/Eskenazi Health, Indianapolis, Indiana; Indiana university/Eskenazi Health, Indianapolis, Indiana

## Abstract

**Introduction:**

Pharmacists in our burn center have historically assisted with discharges and transitions of care for patients sent home or to a facility. Despite these efforts, pharmacists were not formally involved in managing burn clinic patients. Collaborative Drug Therapy Management (CDTM) protocols allow pharmacists working within a defined context to independently assume responsibility for direct patient care activities. The goal of implementing this model in our clinic was to improve access to care and streamline management of pharmacologic issues. The objective of our study was to assess the impact of a clinical pharmacist on medication management in an adult burn clinic via a CDTM protocol.

**Methods:**

The CDTM protocol allows pharmacists to independently manage the following disease states via in-person or telephone visits: pain, agitation, delirium, insomnia, venous thromboembolism, skin/soft tissue infections, and hypermetabolic complications. All treatment decisions, interventions, and education are documented in the electronic record. “Incident-to” billing is completed at Level 99211. All pharmacist visits between 1/1/22-9/21/22 were included for review in the study. Demographics were reported for unique patients, and interventions were included from each visit for patients with multiple pharmacist visits.

**Results:**

A total of 19 patients were seen at 39 visits with a clinical pharmacist during the study. Patients were mostly males (84%) with a mean (SD) age of 45 (18) years. Majority of patients were in-state (95%), with 11 (58%) being from an outside county. Patients were seen for a median (IQR) of 2 (1,2) visits each. Additional interventions were made in 12 patients (63%) at 19 visits (49%), including medication reconciliation [16 (41%)], medications ordered [14 (36%)], labs ordered [2 (5%)], referrals placed [1 (3%)], and allergies addressed [1 (3%)]. At applicable visits, patients had a median (IQR) of 2 (1,2) interventions made or 2 (1, 3) medications ordered.

**Conclusions:**

Historically, pharmacists were only involved with clinic patients when issues arose, serving in an “as-needed” capacity. Pharmacists are now able to proactively help with medication reconciliation, medication prescribing, ordering labs, and placing referrals. Implementing a CDTM protocol has allowed our pharmacists to become more formally involved in post-discharge follow-up and managing ambulatory burn patients.

**Applicability of Research to Practice:**

To our knowledge, ours is the first burn center to implement a Clinical Pharmacist CDTM Protocol, which may serve as a framework for others. Future directions include continuing to track data for adherence, medication access, billing/reimbursement, and clinical outcomes.

